# Predictors for Smoking Cessation with Acupuncture in a Hong Kong Population

**DOI:** 10.1155/2015/189694

**Published:** 2015-08-11

**Authors:** Zhao Liu, Jin-sheng Yang, Yuan Wu, Ou Zhang, Min Chen, Ling-ling Huang, Xiu-qing He, Guan-yi Wu, Ying-ying Wang

**Affiliations:** ^1^Institute of Acupuncture and Moxibustion, China Academy of Chinese Medical Sciences, Beijing, China; ^2^Hong Kong Pok Oi Hospital, Hong Kong

## Abstract

*Background*. Observational studies of smoking cessation with acupuncture have been reported widely; however, few researchers have focused on its predictors. *Objective*. This paper attempts to explore the predictors for smoking cessation with acupuncture in a Hong Kong population, aiming to provide references for clinical treatment in the future. *Methods*. We performed a secondary analysis of data from our observational study “Acupuncture for Smoking Cessation (2011–2014)” in Hong Kong. A total of 23 indexes were selected as possible predictors, and study participants with complete information of 23 indexes were included. By taking 8-week and 52-week smoking cessation results as dependent variables, binary logistic regression method was used to identify the predictors. Additionally, based on an M5P decision-tree algorithm, an equation of “successful rate of smoking cessation with acupuncture” was calculated. *Results*. (1) 2,051 study participants were included in total. (2) According to the results of binary logistic regression, variables including treatment location, total number of acupuncture sessions received, and whether the study participants received at least 6 sessions of acupuncture were taken as the short-term predictors; gender, treatment location, Fagerstrom Test for Nicotine Dependence (FTND), and total number of acupuncture sessions received were taken as the long-term predictors. (3) According to study participants' FTND, treatment location, and number of cigarettes smoked/day, the equation of “successful rate of smoking cessation with acupuncture” was established. *Conclusion*. Receiving sufficient and qualified acupuncture is the leading factor for short-term smoking cessation with acupuncture, whereas individual factors and smoking background play a more important role in long-term smoking cessation with acupuncture.

## 1. Introduction

Smoking is believed to be a serious public health problem around the world. In Hong Kong Special Administrative Region (HKSAR), smoking is the leading factor for death and premature death, and the top-5 diseases that would lead to death are all related with smoking [1]. As shown in the government report of HKSAR [2], 648,800 smokers are considered as daily smokers; smoking causes more than 6,900 cases of death each year, including 1,324 cases of passive smoking. Additionally, the health care spending due to smoking and passive smoking is up to 5.3 billion HKD every year [3]. As a result, the HKSAR Heath Department established the Tobacco Control Office in February, 2001 [4], and various smoking cessation services, such as hotlines, clinic services and an online interaction center, and psychological counseling and medication are currently provided for smokers attempting to quit.

In order to make a “non-smoking life” proposed by HKSAR government, with the support of the Tobacco Control Office of HKSAR Heath Department and the cooperation of Hong Kong Pok Oi Hospital, the Institute of Acupuncture and Moxibustion, China Academy of Chinese Medical Sciences, performed a observational study, namely, “Acupuncture for Smoking Cessation” from 2011 to 2014 [5]. In this observational study, we applied traditional Chinese medicine (TCM) mobile medical vehicles to provide service for smoking cessation with acupuncture, which covered Hong Kong Kowloon and New Territories. Meanwhile, night-time service was set up, aiming to help study participants to comprehensively quit tobacco dependence.

However, due to the individual differences of smokers, if there is no specific strategy for smoking cessation, it is highly unlikely that smokers will be able to quit tobacco dependence, in particular, with acupuncture. This indicates that if we could find the predictors for smoking cessation with acupuncture, specific strategies could be able to be established and smokers would be more likely to quit smoking. Previous research [6–8] indicated that older age, male, a reduced number of cigarettes smoked/day, more attempts at quitting smoking, and being married are essential indicators to predict whether successful smoking cessation can be achieved. Yet, these results were mostly based on nicotine replacement therapy (NRT).

This study is a secondary analysis of data from study participants who attended our observational study [5] in HKSAR and aims to explore the predictors for smoking cessation with acupuncture. It hopefully provides references for smoking quitters and clinical doctors in future intervention.

## 2. Data and Methods

### 2.1. Data Sources

This study analyzed the study participants who participated in the observational study, namely, “Acupuncture for Smoking Cessation” supported by the Tobacco Control Office of HKSAR government and Hong Kong Pok Oi Hospital. This observational study recruited motivated daily smoking quitters aged from 18 to 75 years. After signing an informed consent form, study participants were treated with acupuncture. The acupuncture treatment was given with 0.25 mm × 25 mm disposable needles at Baihui (GV 20), Yintang (GV 29), Lieque (LU 7), Hegu (LI 4), Neiguan (PC 6), Sanyinjiao (SP 6), and Taichong (LV 3). The needles were retained for 20 minutes during each treatment. The intervention of smoking cessation was given three times a week for 8 weeks, and the smoking abstinence rates at 8 weeks and 52 weeks were taken as trial outcomes. Part of this trial was published elsewhere [5], and the method section will not be discussed further.

### 2.2. Data Collection and Summary

All the data were collected and analyzed by two independent researchers from the Institute of Acupuncture and Moxibustion, China Academy of Chinese Medical Sciences. When there were disagreements, they were resolved by discussion between the two independent researchers to make sure the data collection was accurate and objective.

Based on previous studies [9–14], twenty-three predictors in the data were taken as possible predictors. They were

1. demographic data, including gender, age, education, residential zone, and treatment location;
2. smoking background, including smoking history (years), number of cigarettes smoked per day, Fagerstrom Test for Nicotine Dependence (FTND), number of previous quit attempts, and expired carbon monoxide (CO) before acupuncture;
3. quitting reason and motivation, including quitting confidence (presented with 0 to 10 points), quitting preparation (presented with 0 to 10 points), and reasons to select acupuncture, due to their own health or not, due to health of family member or not, due to good looking or not, persuaded by someone or not, and due to money saving or not;
4. treatment compliance, including total number of acupuncture sessions received, whether the participants received at least 6 sessions of acupuncture, and whether the participants received at least 6 sessions of acupuncture within first month;
5. smoking cessation results, including 8-week and 52-week smoking cessation results.

The study participants with complete information of 23 indexes above were included in this study to analyze the relationship between possible predictors and smoking cessation results.

### 2.3. Statistical Analysis

All the data were analyzed with SPSS 19.0 software. The enumeration data was expressed with frequency and percentage, and measurement data was shown with mean ± standard deviation (*X* ± *S*). Binary logistic regression was adopted to analyze the relationship between possible predictors and smoking cessation results and shown as odds ratio (95% confidence interval). Weka Explorer 3.6 software was applied to perform an M5P decision-tree algorithm to propose the equation of “successful rate of smoking cessation with acupuncture.” Statistical significance for all the analysis was evaluated at *P* < 0.05.

## 3. Results

**Figure 1:**
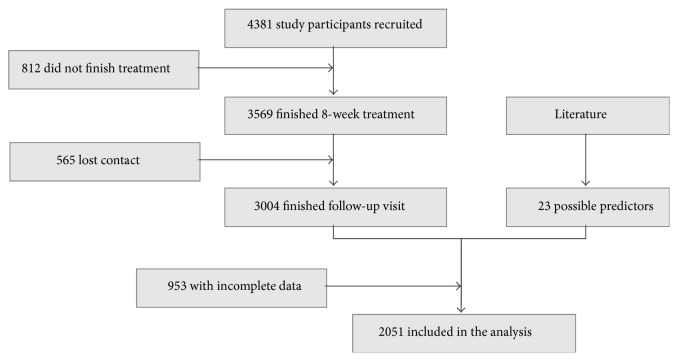
Study flow chart.

The observational study “Acupuncture for Smoking Cessation” recruited 4,381 study participants in total, among which 2,051 study participants with complete information were included in this study. Their general data was shown in Table 1. The study flow chart could be seen in Figure 1.

### 3.1. Baseline Data of Smoking Cessation with Acupuncture

We collected baseline data regarding demographic data, smoking background, quitting reason and motivation, treatment compliance, and smoking cessation results. The characteristics of the study population were presented in Table 1.

### 3.2. Binary Logistic Regression of Smoking Cessation with Acupuncture

Binary logistic regression was adopted to analyze the relationship between possible predictors and smoking cessation results. The results were shown in Table 2.

Table 2 shows that (1) variables including treatment location, total number of acupuncture sessions, and whether the participants received at least 6 sessions of acupuncture were taken as the short-term predictors; (2) variables including gender, treatment location, FTND, and total number of acupuncture sessions were taken as the long-term predictors.

### 3.3. Equation of “Successful Rate of Smoking Cessation with Acupuncture”

**Figure 2:**
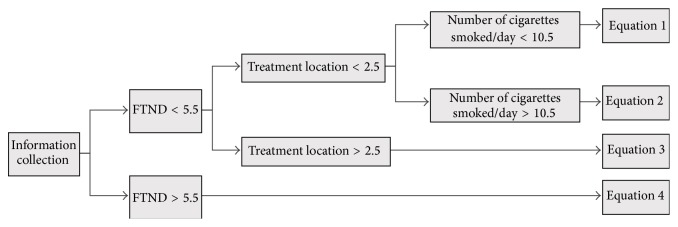
Equation of “successful rate of smoking cessation with acupuncture.”

According to the predictors acquired in Table 2, in order to provide references for further treatment, the equation of “successful rate of smoking cessation with acupuncture” was calculated. By taking 8-week (end of treatment) smoking cessation results as the dependent variable and other variables as independent variables, M5P decision-tree algorithm was adopted to calculate the equation. Results were shown in Figure 2.

Figure 2 showed the following.

(1) If a smoking quitter had FTND < 5.5, treatment location < 2.5, and number of cigarettes smoked/day < 10.5, (1) should be used:

(1) Successful  rate  of  smoking  cessation  with  acupuncture=−0.0006×gender+0.0001×residential  zone−0.0003×treatment  location+0.0404×number  of  cigarette  smoked/day−0.0003×FTND−0.0004×attempted  times  of  smoking  cessation−0.0006×expired  CO+0.0006×confidence−0.0022×reasons  to  select  TM  acupuncture+0.1032×due  to  health  of  family  member−0.0018×due  to  good  looking−0.0506.

(2) If a smoking quitter had FTND < 5.5, treatment location < 2.5, and number of cigarette smoked/day > 10.5, (2) should be used:

(2) Successful  rate  of  smoking  cessation  with  acupuncture=−0.0006×gender+0.0001×residential  zone−0.0003×treatment  location+0.0005×number  of  cigarette  smoked/day−0.0003×FTND−0.0055×attempted  times  of  smoking  cessation−0.0393×expired  CO+0.0006×confidence−0.0019×reasons  to  select  TM  acupuncture+0.0015×due  to  health  of  family  member−0.0018×due  to  good  looking+0.371.

(3) If a smoking quitter had FTND < 5.5, treatment location > 2.5, and number of cigarettes smoked/day < 10.5, (3) should be used:

(3) Successful  rate  of  smoking  cessation  with  acupuncture=−0.0006×gender+0.0072×residential  zone−0.0003×treatment  location−0.0002×number  of  cigarette  smoked/day−0.0003×FTND−0.0049×attempted  times  of  smoking  cessation−0.0006×expired  CO+0.0333×confidence−0.0007×reasons  to  select  TM  acupuncture+0.0016×due  to  health  of  family  member−0.0019×due  to  good  looking+0.0691.

(4) If a smoking quitter had FTND > 5.5, (4) should be used:

(4) Successful  rate  of  smoking  cessation  with  acupuncture=−0.07×gender−0.0045×treatment  location+0.0037×smoking  duration−0.0154×FTND+0.0001×confidence+0.0087×preparation−0.0001×reasons  to  select  TM  acupuncture+0.0004×due  to  health  of  family  member+0.0309×due  to  good  looking+0.0878.

The relative absolute error of this model was 19.6554%, and root relative squared error was 61.4666%, indicating the model was acceptable.

## 4. Discussions

This study aimed to explore predictors for smoking cessation with acupuncture in HKSAR, since these predictors were essential references for establishing an individual acupuncture plan of smoking cessation. As a result, this study made the assumption that predictors for smoking cessation with acupuncture should be divided into two categories: short-time predictors and long-time predictors, and total number of acupuncture sessions received and treatment location were the most important.

### 4.1. Sufficient and Qualified Acupuncture Treatment

According to the logistic regression, the OR of “total sessions of acupuncture” was 1.171, and that of “whether the participants finished at least 6 sessions of acupuncture” was 5.942. This indicated that the more acupuncture treatment received, the more possibility to quit tobacco dependence with acupuncture.

Different therapeutic environments can have a great influence on treatment results [15, 16], especially for acupuncture because different levels of technique would easily lead to different results. We established 20 different clinics of smoking cessation with acupuncture in our study; however, the abstinence rates in several treatment locations were significantly different from that in other treatment locations. This meant that the quality of acupuncture treatment and attitudes of service would have an essential influence on the abstinence rate. In this study we also found that several participants might give up treatment due to the traffic and the long distance between treatment and home, although we used medical mobile vehicles to provide smoking cessation service into communities. The deeper reason was that they did not have necessary knowledge of acupuncture, leading to easy give-up and insufficient treatment. So it was recommended that the popularization of acupuncture for smoking cessation should be strengthened, including performing community teaching, sending out advertisement materials, introducing the theory and advantage of acupuncture, and presenting successful examples of smoking quitting.

Thus, receiving sufficient and qualified acupuncture is the leading factor for smoking cessation with acupuncture, regardless of short term or long term.

### 4.2. Specificity of Gender of FTND

While female smokers have less daily smoking and nicotine dependence, their success rate of smoking cessation was lower than that of male [17–19]. The reasons could be associated with females usually having more negative feelings towards acupuncture, such as fear and resistance [20, 21], so during the smoking cessation with acupuncture, it was necessary to provide female smoking quitters with more attention and care than usual, such as introducing acupuncture knowledge, how to control weight after smoking cessation, and how to acquire support from family and friends, hoping to effectively improve female long-term abstinence rates [22].

FTND refers to Fagerstrom Test for Nicotine Dependence, which is used to measure the severity of nicotine dependence [23]. FTND > 7 is believed to be severe nicotine dependence. Consistent with studies [24–27], we believed that smoking quitters with higher FTND would need more intensive individual treatment to quit tobacco dependence.

### 4.3. Secondary Predictors

Firstly, education background is considered as an important factor in predicting whether one can quit smoking or not. This meant smoking quitters with higher education are more likely to become free of tobacco dependence. The possible reason could be that [28, 29] higher education can lead to more understanding of the damage of smoking on health and environment conditions, which is thus more motivation for smoking cessation. Interestingly, this research found that more education did not lead to more smoking quitting. This was probably because the education of study participants in this study was considerably low.

Secondly, this study also discussed the motivations of smoking cessation. It is usually believed [30, 31] that smoking cessation is promoted by motivation, but the motivation varies from person to person. Someone may quit smoking due to health problems or economic, family, or work reasons. So motivation is unlikely to be a predictor, which is in accordance with other researches [32].

Finally, older age might lead to smoking cessation [33]. Research in Korea [10] concluded that smoking quitters with age > 65 were often accompanied with basic diseases, so their success rate for quitting smoking was higher. Our research included study participants aged between 31 and 50. While they claimed they quit smoking due to health problems, their motivation was lower than that of participants with a higher age. In this sense, age is not considered as a predictor in this research, but it still has valuable clinical significance.

### 4.4. Prediction Equation

We proposed an equation which is “successful rate of smoking cessation with acupuncture” in the study, because we would like to provide a prediction guide for clinical doctors, so they could assess the conditions of smoking quitters and establish an acupuncture strategy, and if clinical doctors found the successful rate of a smoker was low, then clinical doctors needed to use, besides acupuncture, stronger intervention such as NRT and psychological counseling to help him to quit.

### 4.5. Limitations

This study had several limitations. Firstly, we had 20 different clinics in our study. We initially hoped we could recruit more smokers but these 20 clinics had different treatment quality, which complicated the results. Secondly, the result of smoking cessation was based on self-report without biochemical verification. This could lead to an overestimation of success rates. Thirdly, the use of this equation was limited because some variables such as residential zone and treatment location were exclusive in this study, but we hoped further study could develop a more general one.

## 5. Conclusion

We conclude that receiving sufficient and qualified acupuncture is the leading factor for short-term smoking cessation with acupuncture, while individual factors and smoking background play a more important role in long-term smoking cessation with acupuncture.

## Figures and Tables

**Table 1:** Characteristics of smoking cessation with acupuncture.

Category	Variables	Results
Demographic data	Gender	
	Male, *n* (%)	1368 (66.70%)
	Female, *n* (%)	683 (33.30%)
	Age (years)	43.83 ± 12.33
	Education level	
	Below elementary school, *n* (%)	95 (4.63%)
	Elementary school, *n* (%)	153 (7.46%)
	Middle school, *n* (%)	1264 (61.63%)
	Precollege, *n* (%)	276 (13.46%)
	College or above, *n* (%)	263 (12.82%)
	Residential zone (location number)	8.85 ± 4.75
	Treatment location (location number)	2.85 ± 4.37

Smoking background	Smoking history (years)	25.49 ± 11.87
	Number of cigarettes smoked/day (cigarette)	17.57 ± 8.27
	FTND (points)	5.29 ± 2.32
	Number of previous quit attempts	
	0, *n* (%)	381 (18.58%)
	1, *n* (%)	593 (28.91%)
	2–5, *n* (%)	945 (46.08%)
	6–10, *n* (%)	68 (3.32%)
	10 and above, *n* (%)	64 (3.31)
	Expired carbon monoxide before acupuncture (ppm)	15.09 ± 9.45

Reasons and motivation	Quitting confidence (points)	7.44 ± 1.84
	Quitting preparation (points)	8.12 ± 1.72
	Reason to select acupuncture	
	Advertisement, *n* (%)	1031 (50.27%)
	Received before, *n* (%)	132 (6.44%)
	Trying new method, *n* (%)	577 (28.13%)
	Believing in acupuncture, *n* (%)	311 (15.16%)
	Due to their own health	
	No, *n* (%)	371 (18.09%)
	Yes, *n* (%)	1680 (81.91%)
	Due to health of family member	
	No, *n* (%)	1210 (59.00%)
	Yes, *n* (%)	841 (41.00%)
	Due to good looking	
	No, *n* (%)	1722 (83.96%)
	Yes, *n* (%)	329 (16.04%)
	Persuaded by someone	
	No, *n* (%)	1080 (52.66%)
	Yes, *n* (%)	971 (47.34%)
	Due to money saving	
	No, *n* (%)	1381 (67.33%)
	Yes, *n* (%)	670 (32.67%)

Treatment compliance	Total number of acupuncture sessions received (treatment times)	4.89 ± 2.85
	Whether the participants finished 6 sessions within first month	
	No, *n* (%)	954 (46.51%)
	Yes, *n* (%)	1097 (53.49%)
	Whether the participants finished at least 6 sessions of acupuncture	
	No, *n* (%)	898 (43.78%)
	Yes, *n* (%)	1153 (56.22%)

Treatment result	8 weeks	
	Fail, *n* (%)	1363 (64.46%)
	Success, *n* (%)	688 (33.54%)
	52 weeks	
	Fail, *n* (%)	1703 (83.03%)
	Success, *n* (%)	348 (16.97%)

**Table 2:** Binary logistic regression evaluating the relationship between possible predictors and smoking cessation results.

Category	Variables	8 weeks		52 weeks	
		*P*	OR (95% CI)	*P*	OR (95% CI)
Demographic data	Gender	0.056	0.711 (0.501–1.008)	**0.023**	0.710 (0.528–0.955)
	Age	0.472	0.991 (0.967–1.016)	0.577	0.994 (0.973–1.016)
	Education	0.207	0.900 (0.764–1.060)	0.582	0.960 (0.832–1.109)
	Residential zone	0.661	1.007 (0.977–1.037)	0.052	1.026 (1.000–1.053)
	Treatment location	**0.018**	1.039 (1.007–1.072)	**0.001**	0.944 (0.914–0.976)

Smoking background	Smoking history	0.197	1.017 (0.991–1.043)	0.449	1.009 (0.986–1.032)
	Number of cigarettes smoked/day	0.149	0.982 (0.958–1.006)	0.093	0.982 (0.961–1.003)
	FTND	0.766	0.988 (0.912–1.070)	**0.002**	0.899 (0.839–0.963)
	Number of previous quit attempts	0.102	0.984 (0.965–1.003)	0.102	0.987 (0.971–1.003)
	Expired CO	0.498	1.057 (0.901–1.240)	0.327	0.935 (0.817–1.070)

Reasons and motivation	Quitting confidence	0.510	1.035 (0.935–1.145)	0.225	1.056 (0.967–1.152)
	Quitting preparation	0.112	1.095 (0.979–1.227)	0.961	0.998 (0.908–1.096)
	Reasons to select acupuncture	0.141	1.095 (0.970–1.236)	0.151	0.926 (0.833–1.029)
	Due to health of his own	0.366	0.842 (0.579–1.223)	0.206	0.815 (0.594–1.119)
	Due to health of family member	0.225	0.832 (0.619–1.120)	0.139	1.207 (0.941–1.549)
	Due to good looking	0.391	1.203 (0.788–1.836)	0.224	0.789 (0.538–1.156)
	Due to persuasion	0.267	0.845 (0.627–1.138)	0.847	1.025 (0.795–1.322)
	Due to money saving	0.772	0.944 (0.686–1.298)	0.210	1.187 (0.908–1.552)

Treatment condition	Total sessions of acupuncture	**0.000**	1.171 (1.103–1.243)	**0.000**	1.160 (1.098–1.225)
	Whether the participants finished 6 sessions within the first month	0.683	1.159 (0.572–2.349)	0.066	1.962 (0.956–4.023)
	Whether the participants finished at least 6 sessions of acupuncture	**0.000**	5.942 (2.481–14.231)	0.095	0.979 (0.455–2.105)
